# Hirayama disease: the importance of flexion imaging

**DOI:** 10.1259/bjrcr.20210105

**Published:** 2021-09-10

**Authors:** Kieran Kusel, Richard Warne, Rahul Lakshmanan, Michael Mason, Michael Bynevelt, Snehal Shah

**Affiliations:** 1 Department of Radiology, Perth Children’s Hospital, Nedlands, WA, Australia; 2 Neurological Intervention and Imaging Service of Western Australia, Perth Children’s Hospital, Nedlands, WA, Australia; 3 Department of Neurology, Perth Children’s Hospital, Nedlands, WA, Australia

## Abstract

Hirayama disease is a rare cervical myelopathy characterised by asymmetrical upper limb weakness and muscle atrophy in the forearm and hand. MRI of the cervical spine plays an essential role in diagnosis, however, the characteristic findings are often only seen when the patient is imaged with the neck in flexion.

We present a case of a 15-year-old male who presented with left forearm and hand weakness with muscle wasting. An MRI of the cervical spine with the neck in a neutral position demonstrated atrophy of the spinal cord with intrinsic signal abnormality between C5 and C7. Further imaging with the patient’s neck in flexion demonstrated the hallmark features of Hirayama disease. There was anterior displacement of the thecal sac and spinal cord, and an enlarged, crescent-shaped dorsal epidural space which enhanced following i.v. gadolinium administration. The atrophic segment of cord contacted the posterior vertebral bodies when the neck was in full flexion. This case highlights the importance of imaging patients suspected of having this entity with the neck in full flexion in order to make a diagnosis.

## Case report

A 15-year-old male of Asian descent was referred with left forearm and hand weakness as well as associated muscle wasting. Weakness had been present for approximately one year and there was no recalled precipitating trauma or event. His ability to perform fine motor activities had become limited. He had no other significant past medical history. There was no personal or family history of any neurological disorders.

On examination, there was visible muscle wasting to the left forearm, thenar and hypothenar eminences. There was reduced power (4/5) in the median and ulnar nerve distributions, including the intrinsic muscles of the hand. There was no altered sensation. Nerve conduction studies confirmed the presence of median and ulnar neuropathy and electromyography demonstrated denervation of the forearm muscles.

## Imaging findings

**Figure 1.**
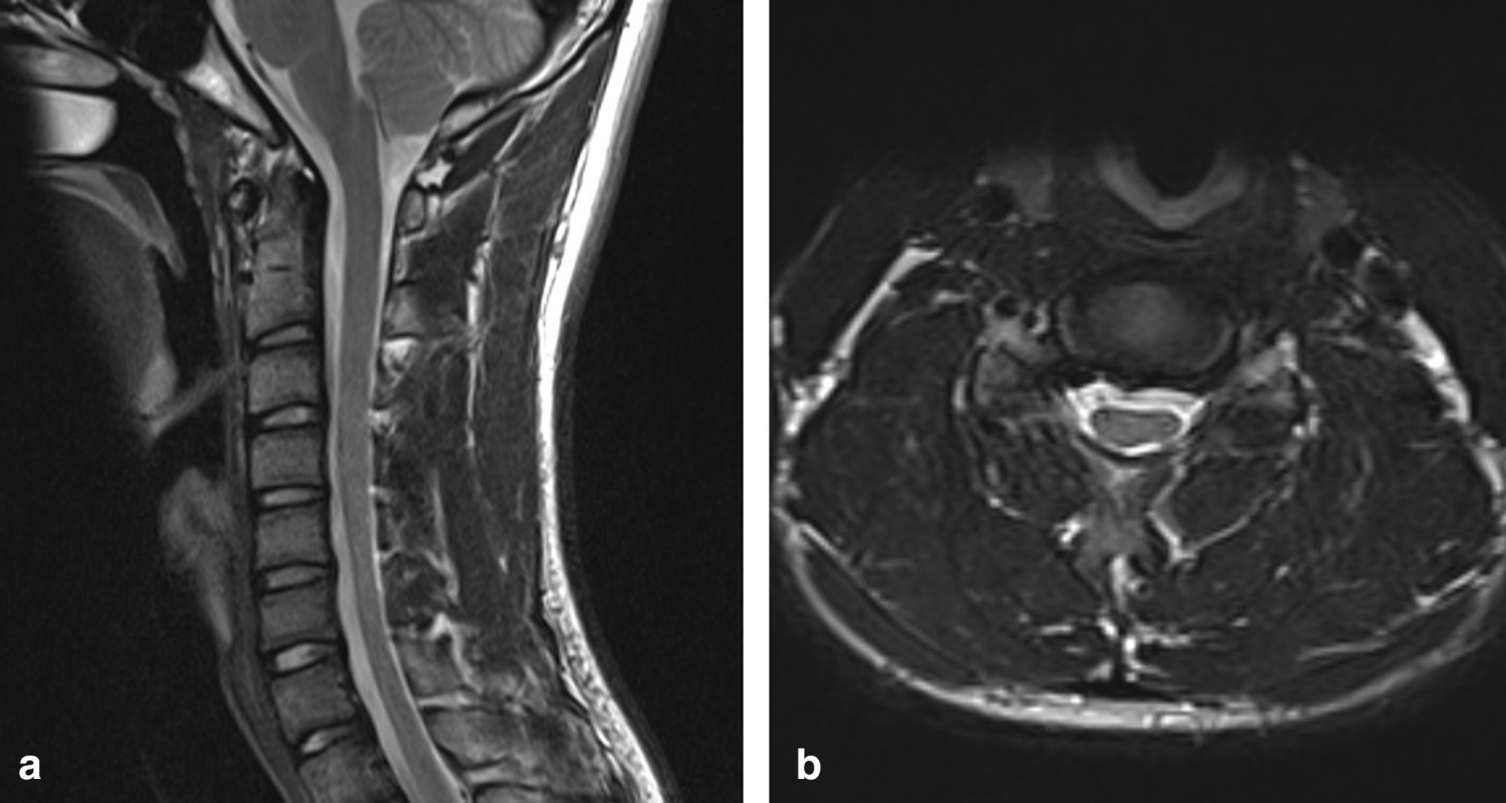
(**a**) T2W sagittal image of the cervical spine in a neutral position demonstrates loss of normal cervical curvature and localised cord atrophy at the C5–C7 level (**b**) T2W axial image in a neutral position at the C6/7 level demonstrates asymmetrical thinning of the cord, more pronounced on the left, with intrinsic cord signal abnormality

An initial MRI of the cervical spine in a supine, neutral position demonstrated mild anteroposterior thinning of the cervical spinal cord between C5 and C7, more pronounced on the left side. There was intrinsic T2 signal hyperintensity in the left hemicord at the C6/7 level (Figure 1).

**Figure 2.**
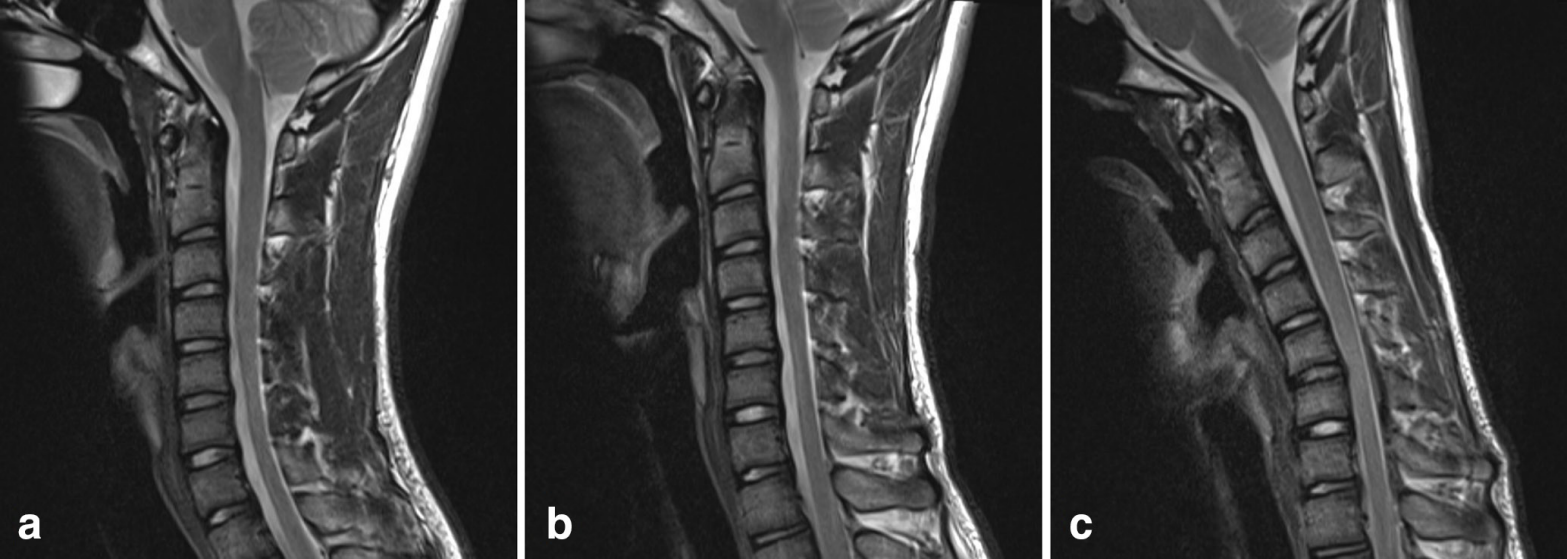
T2W sagittal images of the cervical spine: (**a**) in a neutral position, (**b**) in partial flexion, (**c**) in full flexion. With increasing neck flexion, there is anterior displacement of the thecal sac and spinal cord. In full flexion, the thecal sac contacts the posterior longitudinal ligament and posterior C5–C6 vertebral bodies. There is a corresponding widening of the dorsal epidural space.

**Figure 3.**
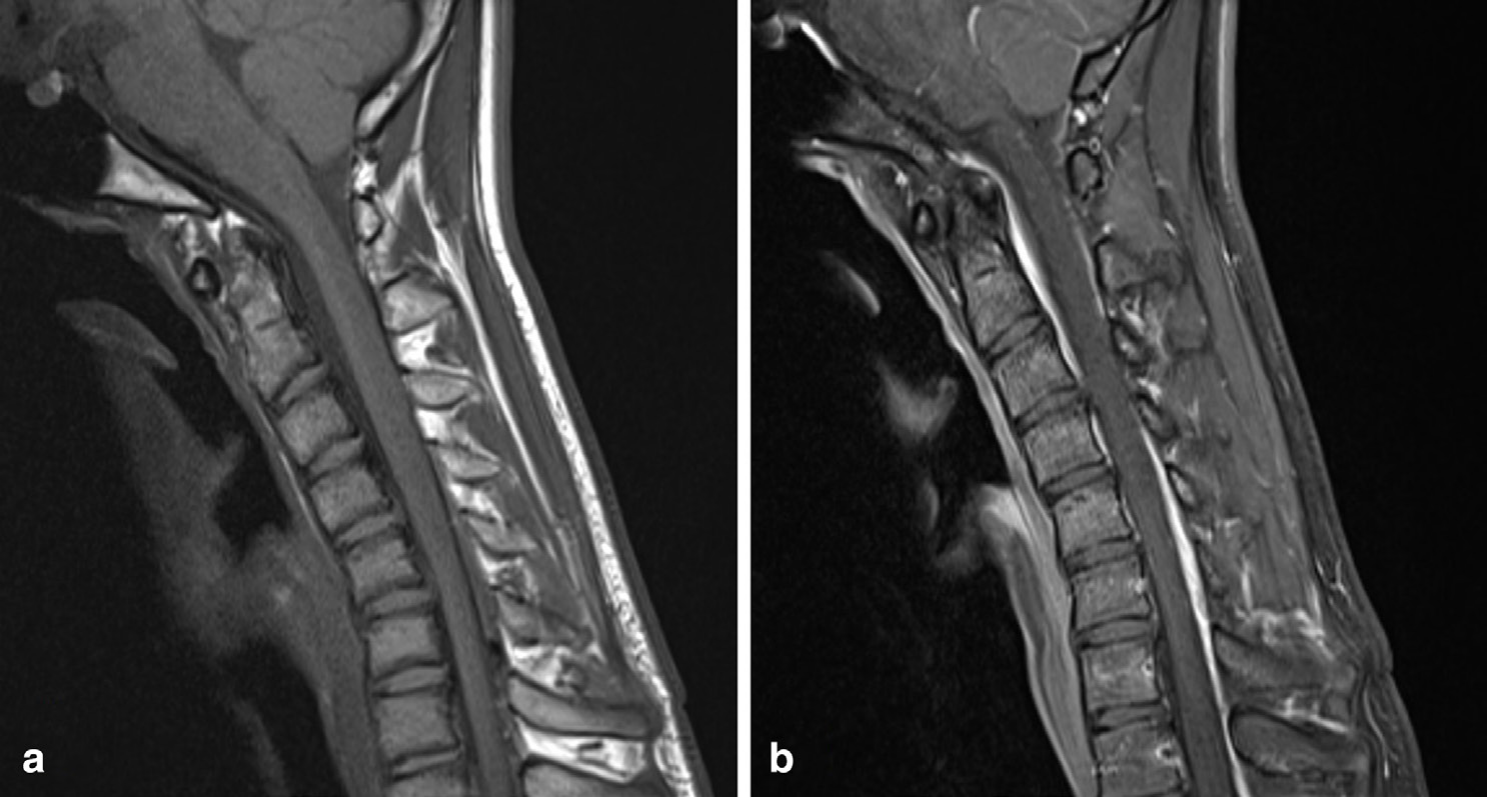
(**a**) T1W sagittal image of the cervical spine with the neck in flexion demonstrates anterior displacement of the thecal sac, an increased laminodural distance and enlarged dorsal epidural space (**b**) Post-contrast T1W sagittal image in the same position demonstrates a crescent-shaped enhancing dorsal epidural space

**Figure 4.**
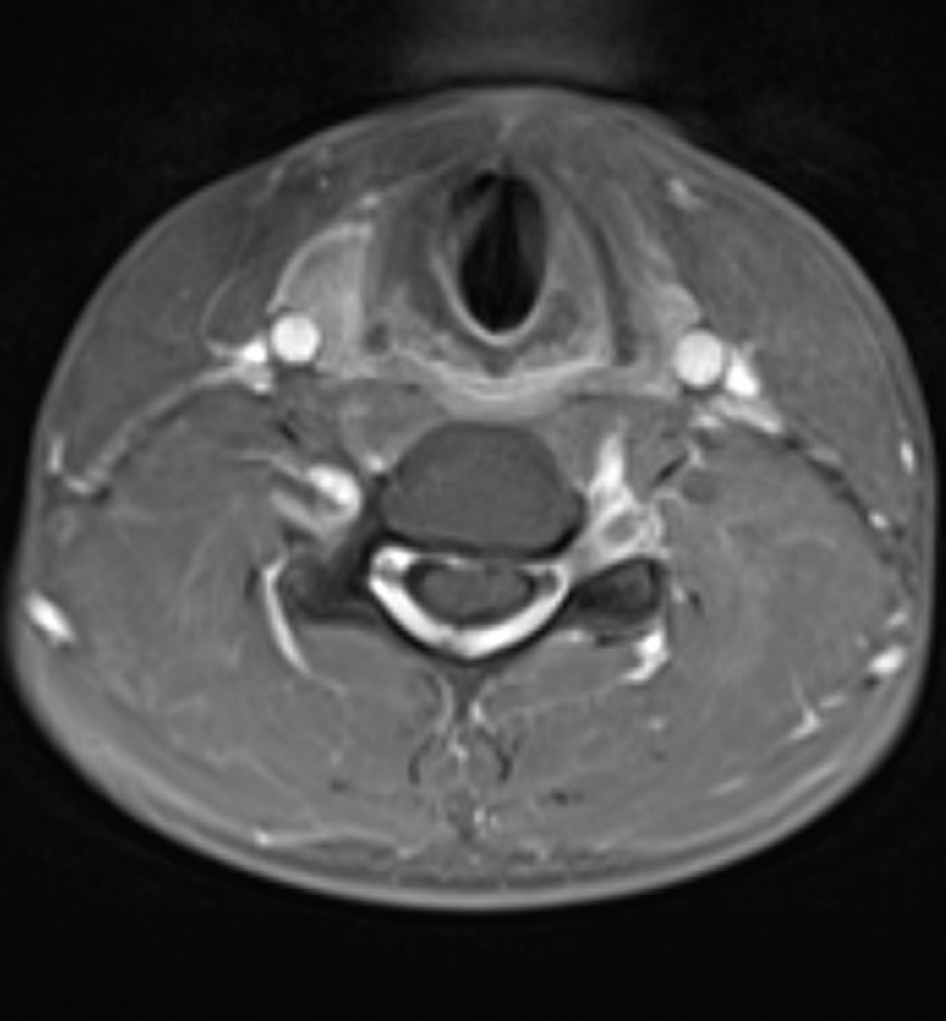
Post-contrast T1W axial image at the C5 level demonstrates anterior displacement of the thecal sac and cervical spinal cord which contact the posterior longitudinal ligament and C5 vertebral body. There is homogeneous enhancement of the widened dorsal epidural space due to the presence of a dilated internal vertebral venous plexus

Further imaging was performed, firstly with the neck in partial flexion and then in full flexion. The patient actively flexed his neck to establish a comfortable position and a support was then placed to maintain this position for the scan. On full flexion, there was ventral displacement of the thecal sac and cord, with widening of the dorsal epidural space (Figure 2). The ventral thecal sac was noted to contact the posterior longitudinal ligament and C5–C6 vertebral bodies. The enlarged, crescent-shaped dorsal epidural space demonstrated avid homogeneous enhancement following i.v. gadolinium administration (Figures 3 and 4).

The patient was referred to the spinal and neurosurgical service and was offered a cervical collar. He was referred to the physiotherapy and occupational therapy services with a view to provide exercises and injury avoidance techniques to reduce the risk of future flexion injuries to the neck.

## Discussion

### Overview

Hirayama disease, also known as juvenile spinal muscular atrophy of the distal upper limb, is a cervical myelopathy characterised by insidious onset upper limb weakness predominantly involving the C7, C8 and T1 myotomes.^1–5^ Weakness is typically unilateral. Less commonly when there is bilateral upper limb involvement, symptoms are usually asymmetrical.^5^ Patients develop atrophy of the muscles of the hand and forearm with sparing of the brachioradialis muscle.^1–3^ There is typically no sensory loss or other neurological findings elsewhere in the body.^6^ Finger weakness is generally exacerbated by cold temperatures (a so-called “cold paresis”).^5^

First described by Keizo Hirayama *et al*. in 1959, Hirayama disease is non-progressive and most commonly affects males between 15 and 25 years of age.^1–5^ Its highest prevalence is in the Asian population, with large case numbers reported in Japan, China and India.^4-6^ There are, however, an increasing number of cases being reported in North America, Europe and Australia, thought to be largely due to an increasing Asian population throughout the rest of the world.^5,7–9^

### Pathophysiology

It is thought that Hirayama disease occurs due to discrepant growth of the vertebral column and spinal canal contents.^5,7^ Normally the spinal thecal sac is only anchored cranially to the foramen magnum and posterior surfaces of C2 and C3, and caudally to the coccyx. Further stability is provided by attachment of the dural sheaths of exiting nerve roots to the borders of the neural foramina.^5,7^ The remainder of the dura is suspended in the spinal canal, surrounded by epidural fat, an internal vertebral venous plexus and connective tissue.^5^

When flexing the neck, the slack of the dura is able to compensate for the slight increase in length of the spinal canal.^5^ It is thought, however, that in some individuals, a length discrepancy between a longer vertebral column and a shorter thecal sac may result in the spinal cord and meninges being pulled taut during neck flexion. The thecal sac and cord are displaced anteriorly and pushed against the posterior surface of the vertebral bodies.^7^ This is believed to cause focal ischaemia, presumably due to compression of the anterior cord and anterior spinal artery against the posterior vertebral bodies. This results in chronic microcirculatory changes and/or venous congestion.^1,3,6–8^ The anterior horn cells are more susceptible to ischaemia than surrounding white matter which may result in cellular necrosis.^1,3^ The first autopsy case obtained by Hirayama *et al.* confirmed anteroposterior spinal cord flattening with ischaemic changes and atrophy of the anterior horn cells.^5,8^

A more rapid growth in height seen in males than in females and the growth spurt which occurs in adolescence may account for the young male predominance of Hirayama disease.^5,7^ Peak age of onset is approximately two years after peak longitudinal growth.^5^

### Diagnosis

MRI plays a key role in diagnosis. MRI with the neck in a neutral position may demonstrate localised cord atrophy, flattening of the cervical cord between C5 and C7 and when asymmetric, the more flattened side of the cord corresponds to the affected side. Straightening of the normal cervical lordosis is also seen with intramedullary signal abnormality on *T_2_*-weighted images.^1–3,5,7,10^

MRI with the neck in flexion demonstrates the hallmark signs including loss of attachment of the posterior thecal sac with anterior displacement of the posterior wall of the thecal sac, a crescent-shaped epidural thickening iso/hyperintense to the cord on *T_1_*-weighted images and hyperintense on *T_2_*-weighted images, with enhancement following i.v. gadolinium administration. Small curvilinear flow voids may be seen within the regional thickening. This abnormality disappears when imaging is performed with the neck in neutral position indicating that this represents a temporarily engorged epidural venous plexus. In one study of 45 patients, the mean laminodural width at maximum forward shifting of the posterior thecal sac measured 5.99 mm.^3^ There may be forward shift on flexion imaging in healthy subjects, although this is limited to between 1.0 and 4.2 mm.^3^ Intramedullary signal abnormality is also noted to be at the level of maximum forward shift.^1–3,5–7,10–13^

**Figure 5.**
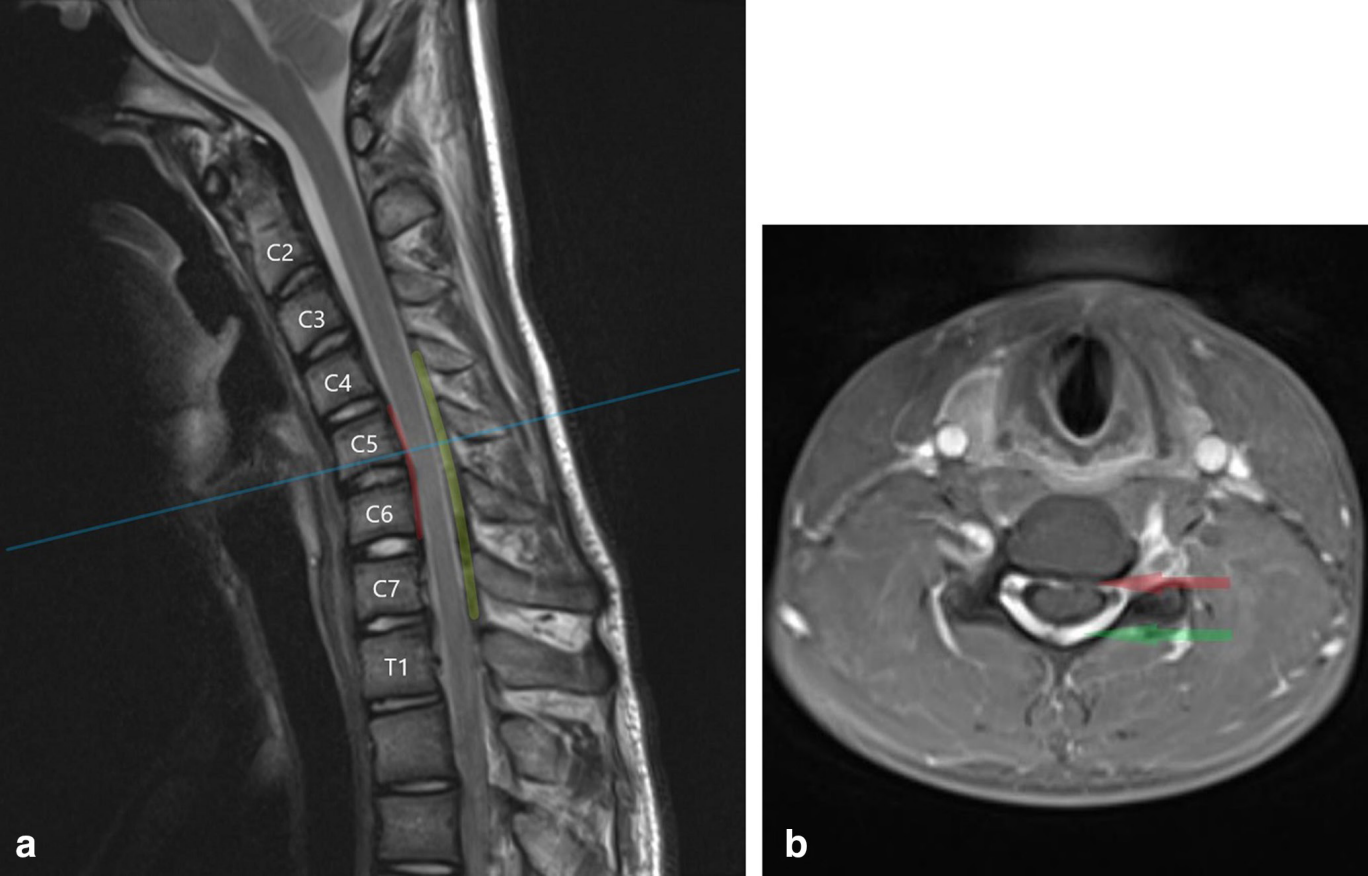
(**a**) T2W sagittal image of the cervical spine in full flexion demonstrates the characteristic findings in Hirayama disease: localised atrophy of the cervical cord between C5 and C7, ventral displacement of the dura and cord which contact the C5 and C6 vertebral bodies (indicated by red line), and an enlarged crescent-shaped dorsal epidural space (indicated in green) (**b**) T1W post-contrast axial image at the C5 level demonstrates anteroposterior flattening of the spinal cord which is displaced ventrally. The anterior dura contacts the C5 vertebral body (red arrow) and there is homogeneous enhancement of the dorsal epidural space (green arrow)

Importantly, MRI of the cervical spine in a neutral position may be normal and imaging with the neck in flexion is essential to demonstrate these hallmark findings.^6,7^ This case highlights the importance of performing imaging with the neck in full flexion. When imaging in partial flexion, these hallmark findings were not readily apparent, and only on full flexion were they clearly demonstrated (Figure 5). Foster *et al.* described a protocol for imaging these patients with routine cervical spine sequences in a neutral, supine position followed by imaging the patient with the neck flexed between 20 and 40 degrees with the head supported by MRI compatible foam pads. They propose that post-gadolinium sagittal and axial fat-suppressed *T_1_*-weighted images should be obtained as a minimum scanning protocol in these patients.^6^

### Treatment

There is currently no treatment to reverse limb weakness, although early recognition and early intervention can stop disease progression.^6,12^ Conservative treatment aimed to reduce neck flexion with a cervical collar is considered a first line treatment.^4,5,7^ It may be worn for 3–4 years until growth spurts are completed.^4,5^ Surgical treatment to prevent cervical flexion with spinal decompression and fusion is reserved for advanced cases or those who demonstrate severe cervical kyphosis on neck flexion imaging.^5–7,10^

## Conclusion

Hirayama disease is a cervical myelopathy typically seen in adolescent males and presents with asymmetrical forearm and hand weakness with muscle wasting. MRI plays an essential role in diagnosis and may demonstrate localised cord atrophy with anteroposterior flattening of the cord and intrinsic cord signal between C5 and C7. However, the hallmark features of this disease may only be seen when imaging the patient with the neck in flexion. Ventral displacement of the thecal sac and cord, and an enlarged, enhancing, crescent-shaped dorsal epidural space are characteristic of this disease. This case highlights these findings and demonstrates the importance of imaging the patient with the neck in full flexion.

## Learning points

- Hirayama disease is a cervical myelopathy characterised by asymmetrical weakness and muscle wasting in the C7, C8 and T1 myotomes.
- MRI of the cervical spine plays an essential role in diagnosis, however, should include imaging with the patient’s neck in a fully flexed position in order to demonstrate the characteristic findings of this disease.
- Forward shifting of the spinal cord and posterior thecal sac with enhancement of the enlarged posterior epidural space (from engorgement of the epidural venous plexus) are characteristic of this disease.
